# Supplementary material for: Multiscale structural complexity assessment of coral reefs using underwater photogrammetry
Source: PLoS One. 2025 Jul 23;20(7):e0318404. doi: 10.1371/journal.pone.0318404 (PMC12286410; doi:10.1371/journal.pone.0318404)
Supplement: S1 File — (DOCX) [file pone.0318404.s001.docx]

| **Site /plot** | **Number of photos** | **Scale error (m)** | **RMS reprojection error** |
| --- | --- | --- | --- |
| Paraiso 1 | 2770 | 0.017 | 0.2477 |
| Paraiso 2 | 3531 | 0.012 | 0.197 |
| Paraiso 3 | 1127 | 0.011 | 0.152 |
| Chankanaab 1 | 1530 | 0.016 | 0.26 |
| Chankanaab 2 | 1301 | 0.015 | 0.24 |
| Chankanaab 3 | 2311 | 0.013 | 0.3 |
| Cardona1 | 790 | 0.01 | 0.25 |
| Cardona2 | 1981 | 0.026 | 0.31 |
| Cardona3 | 741 | 0.04 | 0.33 |
| Yucab1 | 2920 | 0.017 | 0.18 |
| Yucab2 | 2670 | 0.03 | 0.22 |
| Yucab3 | 4077 | 0.01 | 0.15 |
| Francesa1 | 2270 | 0.032 | 0.44 |
| Francesa2 | 1712 | 0.023 | 0.18 |
| Francesa3 | 2652 | 0.023 | 0.25 |
| Colombia1 | 3504 | 0.016 | 0.32 |
| Colombia2 | 2272 | 0.02 | 0.26 |
| Colombia3 | 1950 | 0.03 | 0.39 |

The locations and plots of the coral reef sites evaluated, including the number of datasets per plot, are detailed below. Additionally, the values of the scale error and root mean square (RMS) obtained by post-processing using Agisoft Metashape are provided.
