# Supplementary material for: Multiscale structural complexity assessment of coral reefs using underwater photogrammetry
Source: PLoS One. 2025 Jul 23;20(7):e0318404. doi: 10.1371/journal.pone.0318404 (PMC12286410; doi:10.1371/journal.pone.0318404)
Supplement: S2 File — (DOCX) [file pone.0318404.s002.docx]

List of species identified, coded and classified based on coral morphology in accordance with the Atlantic Gulf Rapid Reef Assessment protocol.

| **Specie** | **Group** | **Code** |
| --- | --- | --- |
| *Agaricia agaricites* | Agariciid | AAGA |
| *Agaricia fragilis* | Agariciid | AFRA |
| *Agaricia humilis* | Agariciid | AHUM |
| *Agaricia lamarcki* | Agariciid | ALAM |
| *Agaricia tenuifolia* | Agariciid | ATEN |
| *Colpophyllia natas* | Meandroid | CNAT |
| *Dendrogyra cylindrus* | Meandroid | DCYL |
| *Dichocoeni stokesii* | Mound & Boulder | DSTO |
| *Diploria Labyrinthiformis* | Meandroid | DLAB |
| *Eusmilia fastigiata* | Flower and solitary | EFAS |
| *Favia fragum* | Mound & Boulder | FFRA |
| *Isophyllia rigida* | Meandroid | IRIG |
| *Madracias decactis* | Branching | MDEC |
| *Mancina areolata* | Meandroid | MARE |
| *Meandrina jacksoni* | Meandroid | MJAC |
| *Meandrina meandrites* | Meandroid | MMEA |
| *Montastraea cavernosa* | Mound & Boulder | MCAV |
| *Mycetophyllia* | Meandroid | MYCE |
| *Mycetophyllia lamarckiana* | Meandroid | MLAM |
| *Orbicella annularis* | Mound & Boulder | OANN |
| *Orbicella Faveolata* | Mound & Boulder | OFAV |
| *Orbicella franksi* | Mound & Boulder | OFRA |
| *Porites astreoides* | Mound & Boulder | PAST |
| *Porites furcata* | Branching | PFUR |
| *Porites porites* | Branching | PPOR |
| *Pseudodiploria clivosa* | Meandroid | PCLI |
| *Pseudodiploria strigosa* | Meandroid | PSEU |
| *Scolymia* | Flower and solitary | SCOL |
| *Siderastrea radians* | Mound & Boulder | SRAD |
| *Siderastrea siderea* | Mound & Boulder | SSID |
| *Solenastrea bournoni* | Mound & Boulder | SBOU |
| *Solenastrea intersepta* | Mound & Boulder | SINT |
