# Supplementary figures and images for: Multiscale structural complexity assessment of coral reefs using underwater photogrammetry
Source: PLoS One. 2025 Jul 23;20(7):e0318404. doi: 10.1371/journal.pone.0318404 (PMC12286410; doi:10.1371/journal.pone.0318404)

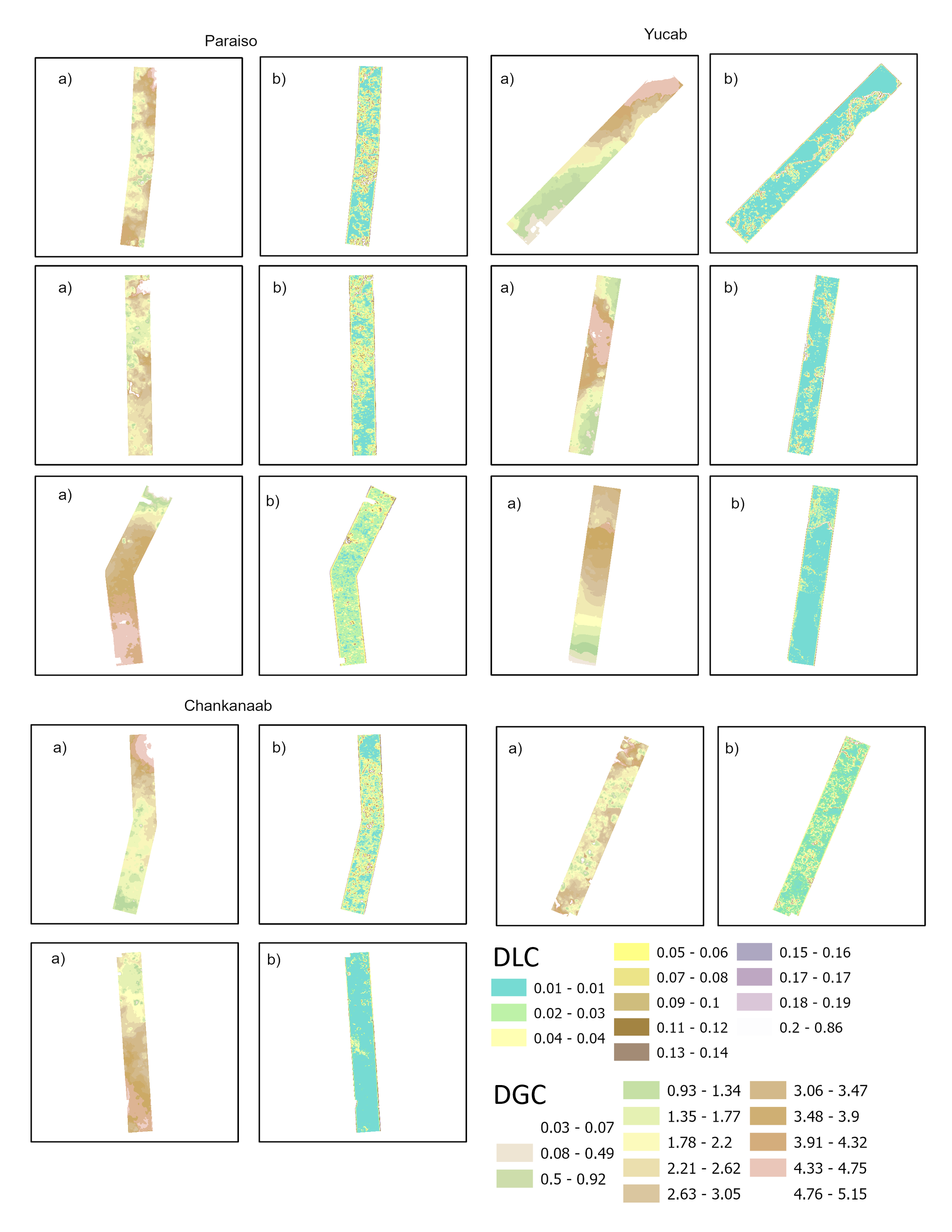


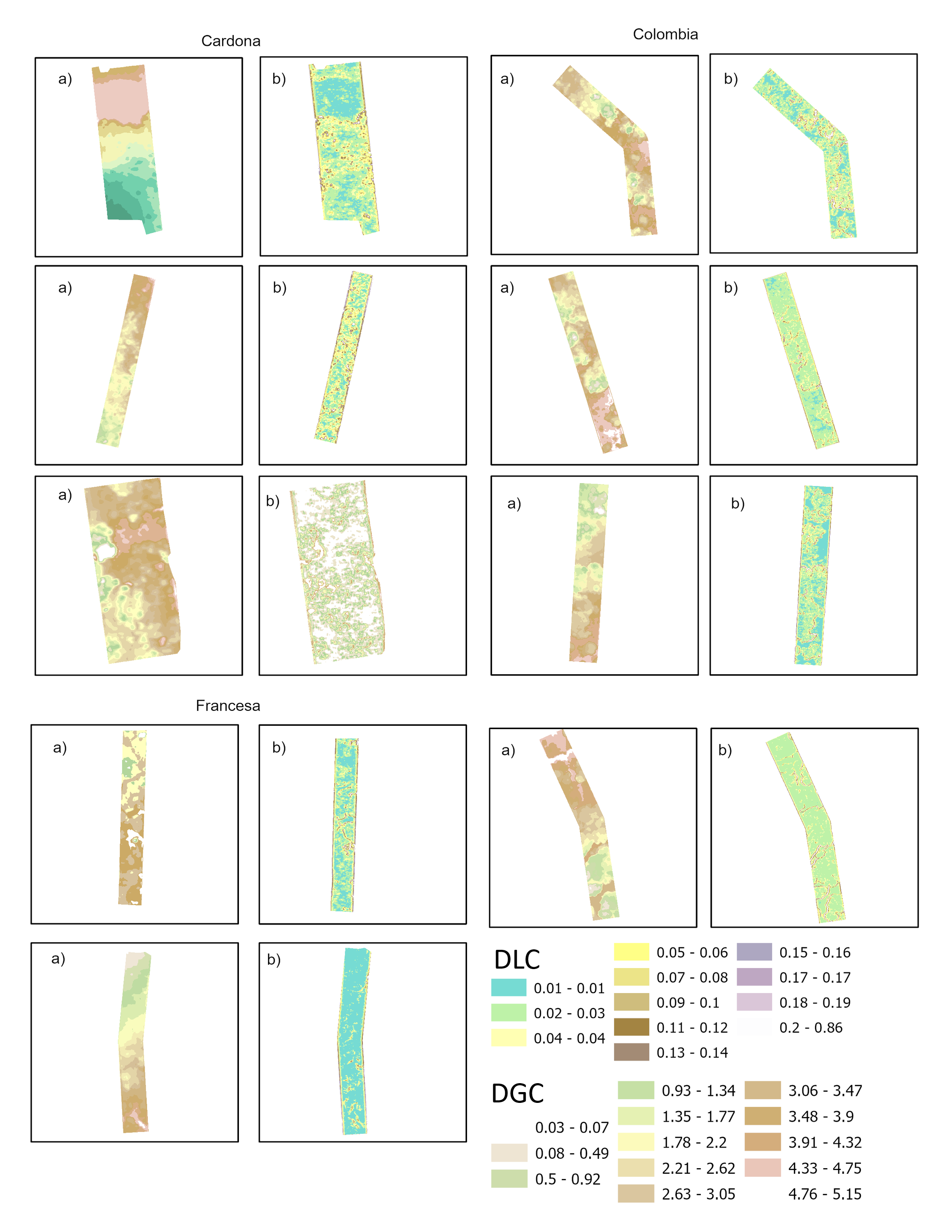

Supplement: S3 File — (DOCX) [file pone.0318404.s003.docx]

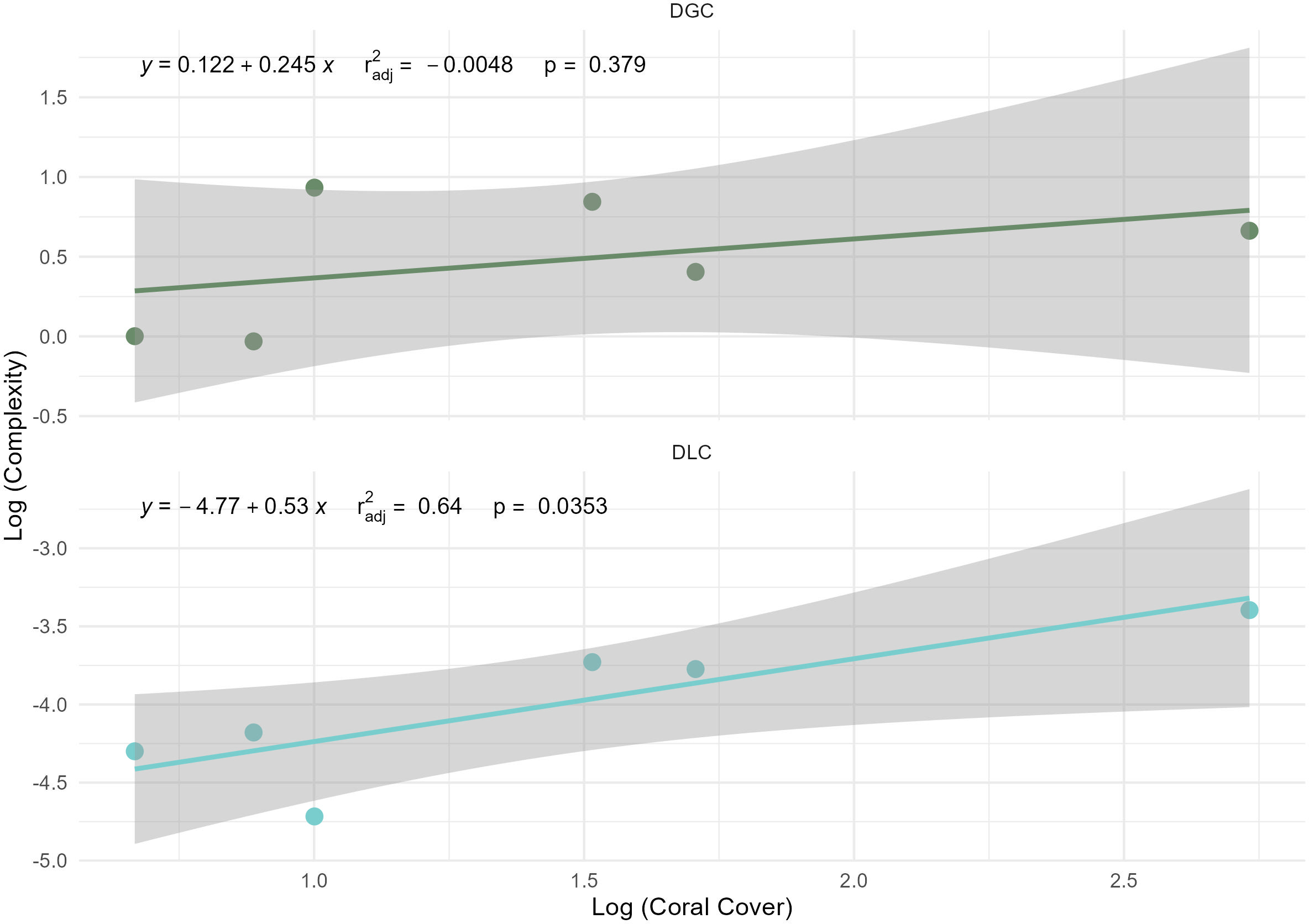

Supplement: S9 File — (TIF) [file pone.0318404.s009.tif]
