# Supplementary material for: Multiscale structural complexity assessment of coral reefs using underwater photogrammetry
Source: PLoS One. 2025 Jul 23;20(7):e0318404. doi: 10.1371/journal.pone.0318404 (PMC12286410; doi:10.1371/journal.pone.0318404)
Supplement: S4 File — (DOCX) [file pone.0318404.s004.docx]

Reef: The name or identifier of the specific coral reef site evaluated.

MW_Statistic: The Mann-Whitney U statistic, which is used to compare two independent samples.

MW_p_value: The p-value from the Mann-Whitney U test.

Reef MW_Statistic MW_p_value

colombia1 7.55149E+12 1

colombia2 4.1242E+12 1

colombia3 2.47921E+12 1

chankanaab1 4.89825E+12 1

chankanaab2 2.33203E+12 1

chankanaab3 1.95238E+13 1

yucab1 3.43925E+13 1

yucab2 6.13338E+12 1

yucab3 1.67975E+13 1

paraiso1 1.77524E+12 1

paraiso2 1.30785E+12 1

paraiso3 1.56857E+12 1

francesa1 2.29273E+11 1

francesa2 6.15943E+11 1

francesa3 6.90409E+12 1

cardona1_1 4.58746E+11 1

cardona1_2 3.65863E+11 1

cardona2 3.46461E+11 1

cardona3 1.90883E+11 1
