# Supplementary material for: Multiscale structural complexity assessment of coral reefs using underwater photogrammetry
Source: PLoS One. 2025 Jul 23;20(7):e0318404. doi: 10.1371/journal.pone.0318404 (PMC12286410; doi:10.1371/journal.pone.0318404)
Supplement: S5 File — (DOCX) [file pone.0318404.s005.docx]

Statistical Analysis of Reef Characteristics: Kruskal-Wallis Test and Dunn's Post-Hoc Comparisons

Kruskal-Wallis rank sum test

data: DGC+DLC by Arrecife

Kruskal-Wallis chi-squared = 4795423, df = 5, p-value < 2.2e-16

Pairwise comparisons using Dunn's all-pairs test

data: DGC+DLC by Reff

Paraiso Chankanaab Yucab Cardona Francesa

Chankanaab <2e-16 - - - -

Yucab <2e-16 <2e-16 - - -

Cardona <2e-16 <2e-16 <2e-16 - -

Francesa <2e-16 <2e-16 <2e-16 <2e-16 -

Colombia <2e-16 <2e-16 <2e-16 <2e-16 <2e-16
