# Supplementary material for: Multiscale structural complexity assessment of coral reefs using underwater photogrammetry
Source: PLoS One. 2025 Jul 23;20(7):e0318404. doi: 10.1371/journal.pone.0318404 (PMC12286410; doi:10.1371/journal.pone.0318404)
Supplement: S6 File — (DOCX) [file pone.0318404.s006.docx]

Kruskal-Wallis rank sum test

data: DGC by Arrecife

Kruskal-Wallis chi-squared = 123861, df = 5, p-value < 2.2e-16

Dunn (1964) Kruskal-Wallis multiple comparison

p-values adjusted with the Bonferroni method.

Comparison Z P.unadj P.adj

1 Cardona - Chankanaab 82.599077 0.000000e+00 0.000000e+00

2 Cardona - Colombia -111.132074 0.000000e+00 0.000000e+00

3 Chankanaab - Colombia -188.230689 0.000000e+00 0.000000e+00

4 Cardona - Francesa -130.633211 0.000000e+00 0.000000e+00

5 Chankanaab - Francesa -198.286738 0.000000e+00 0.000000e+00

6 Colombia - Francesa -51.403801 0.000000e+00 0.000000e+00

7 Cardona - Paraiso 120.062525 0.000000e+00 0.000000e+00

8 Chankanaab - Paraiso 22.472619 7.691882e-112 1.153782e-110

9 Colombia - Paraiso 262.809044 0.000000e+00 0.000000e+00

10 Francesa - Paraiso 257.146701 0.000000e+00 0.000000e+00

11 Cardona - Yucab -123.271573 0.000000e+00 0.000000e+00

12 Chankanaab - Yucab -189.062727 0.000000e+00 0.000000e+00

13 Colombia - Yucab -48.413293 0.000000e+00 0.000000e+00

14 Francesa - Yucab -3.068161 2.153806e-03 3.230709e-02

15 Paraiso - Yucab -240.159802 0.000000e+00 0.000000e+00
