# Supplementary material for: Multiscale structural complexity assessment of coral reefs using underwater photogrammetry
Source: PLoS One. 2025 Jul 23;20(7):e0318404. doi: 10.1371/journal.pone.0318404 (PMC12286410; doi:10.1371/journal.pone.0318404)
Supplement: S7 File — (DOCX) [file pone.0318404.s007.docx]

Kruskal-Wallis rank sum test

data: DLC by Reef

Kruskal-Wallis chi-squared = 73488, df = 5, p-value < 2.2e-16

Dunn (1964) Kruskal-Wallis multiple comparison

p-values adjusted with the Bonferroni method.

Comparison Z P.unadj P.adj

1 Cardona - Chankanaab 61.95534 0.000000e+00 0.000000e+00

2 Cardona - Colombia -83.54159 0.000000e+00 0.000000e+00

3 Chankanaab - Colombia -141.33578 0.000000e+00 0.000000e+00

4 Cardona - Francesa -31.93164 9.715932e-224 1.457390e-222

5 Chankanaab - Francesa -92.22926 0.000000e+00 0.000000e+00

6 Colombia - Francesa 50.22542 0.000000e+00 0.000000e+00

7 Cardona - Paraiso 59.09694 0.000000e+00 0.000000e+00

8 Chankanaab - Paraiso -10.10745 5.119795e-24 7.679692e-23

9 Colombia - Paraiso 157.82175 0.000000e+00 0.000000e+00

10 Francesa - Paraiso 94.49829 0.000000e+00 0.000000e+00

11 Cardona - Yucab 108.84408 0.000000e+00 0.000000e+00

12 Chankanaab - Yucab 33.97562 5.105943e-253 7.658914e-252

13 Colombia - Yucab 218.90475 0.000000e+00 0.000000e+00

14 Francesa - Yucab 146.86019 0.000000e+00 0.000000e+00

15 Paraiso - Yucab 49.99743 0.000000e+00 0.000000e+00
