# Supplementary material for: Multiscale structural complexity assessment of coral reefs using underwater photogrammetry
Source: PLoS One. 2025 Jul 23;20(7):e0318404. doi: 10.1371/journal.pone.0318404 (PMC12286410; doi:10.1371/journal.pone.0318404)
Supplement: S8 File — (DOCX) [file pone.0318404.s008.docx]

Statistical Analysis of Reef Characteristics: Kruskal-Wallis Test and Dunn's Post-Hoc Comparisons

Kruskal-Wallis rank sum test

data: DLC by Group

Kruskal-Wallis chi-squared = 13935, df = 4, p-value < 2.2e-16

Dunn (1964) Kruskal-Wallis multiple comparison

p-values adjusted with the Bonferroni method.

Comparison Z P.unadj P.adj

1 Agariciid - Branching 22.7157916 3.127786e-114 3.127786e-113

2 Agariciid - Flower and Solitary 31.3104696 3.361145e-215 3.361145e-214

3 Branching - Flower and Solitary 23.6896549 4.609282e-124 4.609282e-123

4 Agariciid - Meandroid 47.9158877 0.000000e+00 0.000000e+00

5 Branching - Meandroid 35.7786713 2.370833e-280 2.370833e-279

6 Flower and Solitary - Meandroid 0.6625173 5.076398e-01 1.000000e+00

7 Agariciid - Mound & Bolder 110.3777653 0.000000e+00 0.000000e+00

8 Branching - Mound & Bolder 67.1468960 0.000000e+00 0.000000e+00

9 Flower and Solitary - Mound & Bolder -2.5551915 1.061294e-02 1.061294e-01
