# Supplementary material for: Multiscale structural complexity assessment of coral reefs using underwater photogrammetry
Source: PLoS One. 2025 Jul 23;20(7):e0318404. doi: 10.1371/journal.pone.0318404 (PMC12286410; doi:10.1371/journal.pone.0318404)
Supplement: Attachment — Submitted filename: Answers_to_reviewerV2.docx [file pone.0318404.s012.docx]

Manuscript PONE-D-25-02577R1

**Response to Reviewers**

Dear reviewer, we appreciate your comments and below you will find a detailed answer addressing all the suggestions.

**Reviewer 2.**

Reviewer #2: I would like to thank the authors for the comprehensive revision, and explanation of all changes made. The manuscript has improved significantly in important aspects. However, the following issues still need to be addressed:

Please define “rugosity” more clearly. In some passages you might consider replacing “rugosity” with the broader term like “reef structural complexity” (e.g., lines 18, 35, 153, 299, 319, 370…). In most of the literature, rugosity is defined simply as the ratio of actual surface length to geometric length. And it can be obtained by different methods, for example the chain method, using a depth gauge, or derived from a DEM or point cloud. But some of the metrics you list, such as fractal dimension and VRM mentioned on lines 304-305, are not strictly rugosity. Because these structural complexity metrics are calculated differently, they are not “rugosity metrics.” Therefore, please revise some of the terminology to avoid confusion. Also, calling your new metrics “rugosity” may lead readers to assume they are based on traditional measurement methods, but since you use wavelet-based approach, DLR and DGR represent a different concept. Could you consider giving them new names to avoid confusion with past metrics? Alternatively, please explain why it is appropriate to include the term “rugosity” in DLR and DGR.

Thank you for the suggestions, we agree and have categorized the metrics based on the nature of the calculations to enhance clarity and prevent confusion, lines 73-81. We have also replaced where appliable the term rugosity with either reef structural complexity, structural complexity or complexity. This included modifying the title of the manuscript. Finally, as suggested we have also changed the names of DLR and DGR to Digital Local Complexity (DLC) and Digital Global Complexity (DGC) to describe the two components in our approach.

Line 33-34. “…Our findings indicate that conventional approaches for estimating rugosity undervalue the historical role on assessments and…” Since there are many methods for analyzing reef structural complexity and this study only compared against the chain method, I suggest replacing “conventional approaches” with “the chain method” and replacing “rugosity” to “structural complexity” to better reflect the actual results.

Change performed lines 34-36.

Although the computational principles of your approach differ, if you claim that other methods cannot achieve the same results, it is necessary to analyze more of the previous structural complexity metrics. The paper currently only compares against the earliest chain method, lacks concrete evidence of the shortcomings of other past approaches (as mentioned in lines 79–82). For example, analyses using varied DEM resolutions and different structural complexity metrics can likewise distinguish living biological tissue from the topographic features (see Fukunaga & Burns, 2020; Engleman et al., 2023; Harris et al., 2023). You should include more discussion comparing your method to previous ones; otherwise, the statements in lines 33-34, 299-301, 307-309, 369-371 come across as overinterpretation and should be removed or rephrased.
Fukunaga, A., & Burns, J. H. R. (2020). Metrics of Coral Reef Structural Complexity Extracted from 3D Mesh Models and Digital Elevation Models. Remote Sensing, 12(17), 2676. <https://doi.org/10.3390/rs12172676>
Engleman A, Cox K, Brooke S. (2023). Dead but not forgotten: complexity of Acropora palmata colonies increases with greater composition of dead coral. PeerJ 11:e16101 <https://doi.org/10.7717/peerj.16101>
Harris, D. L., Webster, J. M., Vila-Concejo, A., Duce, S., Leon, J. X., & Hacker, J. (2023). Defining multi-scale surface roughness of a coral reef using a high-resolution LiDAR digital elevation model. Geomorphology, 439, 108852.

We revised and modified the text to better explain the main distinctions between our analysis and other methodologies, such as Vector Roughness Measurement and variation in Digital Elevation Model resolutions, in lines 73-81, 85-89. Furthermore, we improved the discussion in lines 318-325; changed as suggested in a previous comment the lines 34-36, and revised statements in lines 312-313; 386-387.

Figure 2, There is still a lot of empty white space around the figure. Could you adjust the layout or crop the image to show only the relevant area so that the graphic fills more of the space and appears larger and clearer?

We changed the figure 2 by increasing the zoom level.

Figure 6, the grouping categories should be made consistent. For example, “Agariciid” refers to a taxonomic family, whereas “Branching” is a growth form, mixing taxonomic and morphological categories is not academically rigorous. I recommend changing “Agariciid” to the corresponding growth form to maintain consistency, using a scheme like the morphological classification in Zawada et al. (2019). Additionally, it is unclear what “flower” means in “Flower & Solitary”; please replace it with a term that is more commonly used in the literature.
Zawada, K. J., Dornelas, M., & Madin, J. S. (2019). Quantifying coral morphology. Coral Reefs, 38(6), 1281-1292.

We appreciate the suggestions. Although there are alternative morphological categories; we have adopted the grouping categories established by the Atlantic and Gulf Rapid Reef Assessment (AGRRA) initiative [1] lines 157-158. This framework aims to standardize data collection across the region, thereby enhancing the comparability of datasets. We employed these categories to facilitate regional comparisons.

Figure 6, for the three groups on the right that are neither A nor B, please add the label C to indicate that they do not differ significantly from each other but are different from A and B.

We updated the figure as suggested.

For Figure 7, the sample size at each site is very small. If you do not plan to discuss the site‐specific relationship between DLR and coral cover in the content, you could plot all data together to show the overall correlation between DLR and coral cover (and, if needed, use different colors for points from each site). Also, I may have missed it, but Figure 7 is not cited anywhere in the manuscript, please refer to it in the appropriate paragraph.

This figure shows the DLC and coral coverage grouped by morphological groups (fig 7a-e). When grouping the data together, the figure becomes difficult to understand. Therefore, we prefer to maintain it as it is, as it was suggested by the other reviewer. This figure is described in lines 272-280.

For Figure 8, I recommend using a stacked percentage bar chart to more clearly illustrate the composition of each site and the overall group breakdown. Listing individual species in the figure is somewhat distracting; you can instead describe in the text which species belong to each group.

We have changed the figure as recommended and have included a reference to the supplementary information.

1.	Lang JC, Marks KW, Kramer PR, Kramer PA, Ginsburg RN. Protocolos AGRRA version 5.5. Big Pine Key: Ocean Research & Education. 2012.
